# Development and validation of a point-based scoring system for predicting axillary lymph node metastasis and disease outcome in breast cancer using clinicopathological and multiparametric MRI features

**DOI:** 10.1186/s40644-023-00564-9

**Published:** 2023-06-01

**Authors:** Xiaofeng Chen, Zhiqi Yang, Ruibin Huang, Yue Li, Yuting Liao, Guijin Li, Mengzhu Wang, Xiangguang Chen, Zhuozhi Dai, Weixiong Fan

**Affiliations:** 1 Department of Radiology, Meizhou People’s Hospital, Meizhou, 514031 China; 2 Guangdong Provincial Key Laboratory of Precision Medicine and Clinical Translational Research of Hakka Population, Meizhou, 514031 People’s Republic of China; 3 Department of Radiology, The First Affiliated Hospital of Shantou University Medical College, Shantou, 515000 People’s Republic of China; 4 GE Healthcare, Guangzhou, 510623 China; 5 MR Application, Siemens Healthineers, Shanghai, 201318 China; 6 MR Scientific Marketing, Siemens Healthineers, Guangzhou, 510620 China; 7 Department of Radiology, Shantou Central Hospital, Shantou, Guangdong 515041 People’s Republic of China; 8 Department of Radiology, Sun Yat-Sen Memorial Hospital, Sun Yat-Sen University, Guangzhou, 510120 Guangdong China

**Keywords:** Lymphatic metastasis, Prognosis, Breast neoplasms, Multiparametric magnetic resonance imaging, Risk factors

## Abstract

**Background:**

Axillary lymph node (ALN) metastasis is used to select treatment strategies and define the prognosis in breast cancer (BC) patients and is typically assessed using an invasive procedure. Noninvasive, simple, and reliable tools to accurately predict ALN status are desirable. We aimed to develop and validate a point-based scoring system (PSS) for stratifying the ALN metastasis risk of BC based on clinicopathological and quantitative MRI features and to explore its prognostic significance.

**Methods:**

A total of 219 BC patients were evaluated. The clinicopathological and quantitative MRI features of the tumors were collected. A multivariate logistic regression analysis was used to create the PSS. The performance of the models was evaluated using receiver operating characteristic curves, and the area under the curve (AUC) of the models was calculated. Kaplan–Meier curves were used to analyze the survival outcomes.

**Results:**

Clinical features, including the American Joint Committee on Cancer (AJCC) stage, T stage, human epidermal growth factor receptor-2, estrogen receptor, and quantitative MRI features, including maximum tumor diameter, K_ep_, V_e_, and TTP, were identified as risk factors for ALN metastasis and were assigned scores for the PSS. The PSS achieved an AUC of 0.799 in the primary cohort and 0.713 in the validation cohort. The recurrence-free survival (RFS) and overall survival (OS) of the high-risk (> 19.5 points) groups were significantly shorter than those of the low-risk (≤ 19.5 points) groups in the PSS.

**Conclusion:**

PSS could predict the ALN metastasis risk of BC. A PSS greater than 19.5 was demonstrated to be a predictor of short RFS and OS.

**Supplementary Information:**

The online version contains supplementary material available at 10.1186/s40644-023-00564-9.

## Background

Breast cancer (BC) is the most commonly diagnosed cancer among women worldwide and has become the second leading cause of cancer-related death [1]. BC can spread to the regional lymph nodes, primarily to the axillary and internal mammary nodes and subsequently to the medial supraclavicular nodes [2]. Patients with BC frequently experience axillary lymph node (ALN) metastasis, which affects the clinical stage, therapy options, surgical approach, and patient prognosis [1, 2]. Thus, accurate identification of ALN involvement in BC patients is crucial for prognosis and treatment decisions, particularly in the current time of downgrading off axillary surgery. Currently, ALN dissection, sentinel lymph node biopsy (SLNB), and lymph node biopsy before surgery are important procedures for ALN staging. Generally, lymph node biopsy is very important in daily clinical practice for pretreatment staging in patients with suspicious lymph node metastasis. SLNB is the current best standard approach for axillary staging in patients with clinically node-negative BC [3, 4]. ALN dissection is the standard treatment for BC patients with clinically positive nodes, but it might be avoided in patients with negative SLNB, as well as in patients with one or two sentinel lymph node-positivity receiving breast radiation and systemic therapy [5, 6]. However, these are invasive methods with the risk of postoperative complications, especially for ALN dissection [7, 8].

As noninvasive approaches, physical examination, mammography, ultrasonography, and positron emission tomography–computed tomography (PET/CT) are widely used to predict ALN metastasis. However, their abilities to assess the risk of ALN metastasis are limited because of their high false-negative rates and low sensitivity [9–12]. To date, only a few studies have used preoperative MRI to predict ALN metastasis, and these studies have shown that MRI morphological features and enhancement parameters of tumors are correlated with ALN status [13–15]. However, these models have not been widely used due to their complexity in clinical application. Furthermore, the mere identification of ALN metastasis by imaging is insufficient to change the paradigm for axillary surgical strategy [3]. A recently developed and validated nomogram was used to predict the metastasis status of ALNs based on a combination of clinicopathologic and MRI features, in which the developed model performed well for identifying ALN metastasis [10]. However, using the nomogram might be time-consuming and hard to interpret [16]. Therefore, to simplify the eventual clinical application of the predictive model, a point-based scoring system (PSS) was proposed and has been used to predict lymphovascular invasion and differential diagnosis of tumors [16, 17]. In this study, we aimed to use the PSS to predict the risk of ALN metastasis based on clinicopathological and quantitative MRI features and investigated the prognostic effect of the PSS.

## Materials and methods

### Patient characteristics

**Fig. 1:**
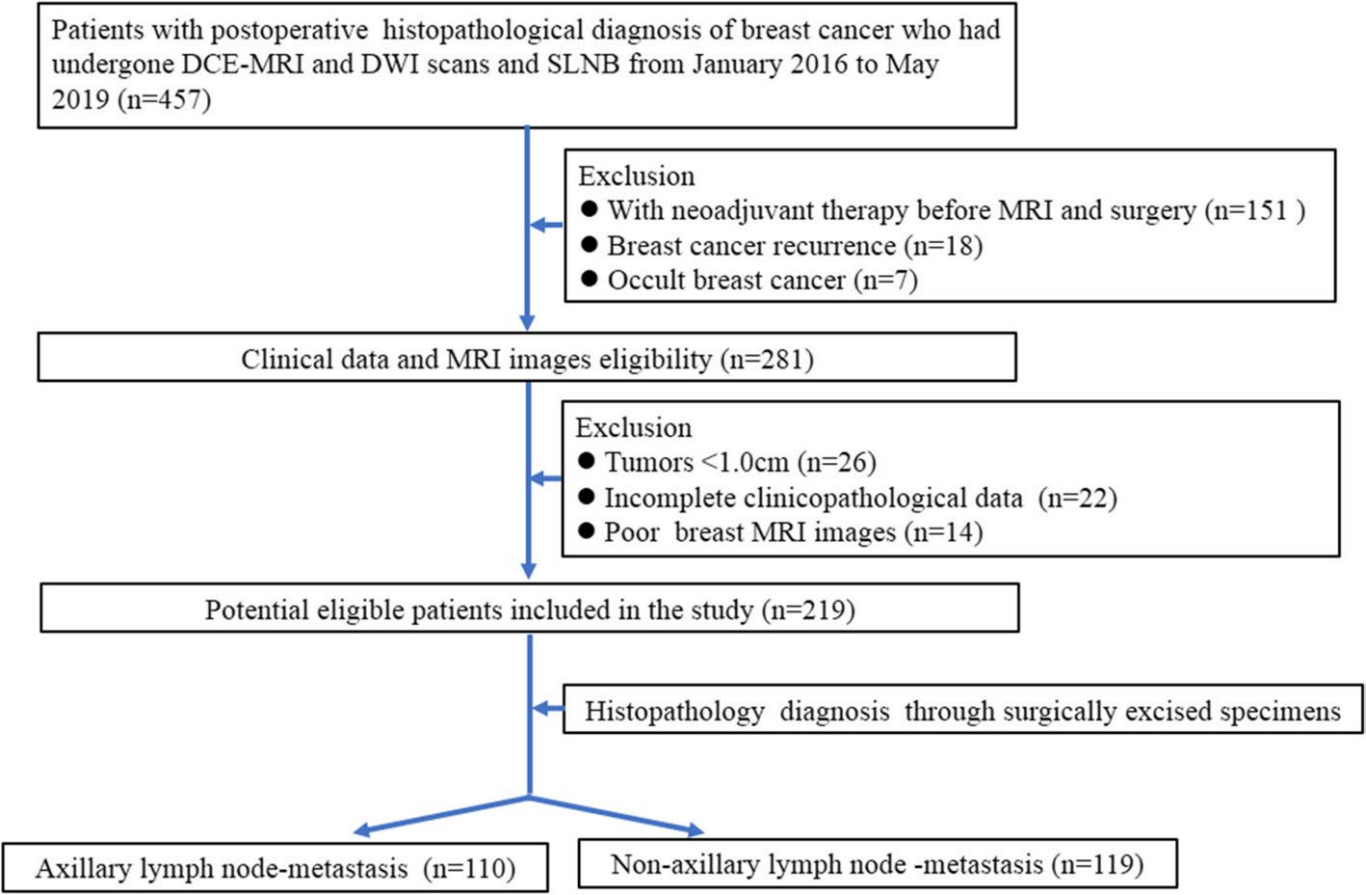
Patient selection flowchart

This retrospective study was approved by the ethics committee of Meizhou People's Hospital and the requirement for written informed consent was waived. We reviewed a total of 457 consecutive female patients with a postoperative histopathological diagnosis of BC who had undergone preoperative dynamic contrast-enhanced MRI (DCE-MRI) and diffusion-weighted imaging (DWI) scans and axillary biopsy or SLNB (complete ALN dissection was performed if the SLNB or axillary biopsy was positive) from January 2016 to May 2019. The patient exclusion criteria were as follows: (1) patients who had received neoadjuvant therapy prior to breast MRI or surgery; (2) patients with breast cancer recurrence; (3) patients with occult breast cancer; (4) patients with a tumor smaller than 1.0 cm;(5) patients with incomplete clinicopathological data; and (6) patients with poor visualization of the tumors on breast MRI. The patient inclusion/exclusion criteria are shown in Fig. 1. Finally, a total of 219 patients were enrolled and randomly divided into a primary cohort and a validation cohort at a ratio of 7:3 according to previous studies [18, 19]. Histological results after surgery were used as the ground truth of ALN metastasis. This study's patients are part of a large retrospective breast MRI database, of which 165 patients have been reported in a previously published study [20]. A previous report evaluated BC receptor status and molecular subtypes; however, this novel study focused on ALN metastasis by developing a new PSS.

### Clinicopathological characteristics

Clinicopathological data, including age, Ki-67 level, human epidermal growth factor receptor-2 (HER-2), progesterone receptor (PR), estrogen receptor (ER), tumor node metastasis (TNM) stage, American Joint Committee on Cancer (AJCC) stage [21], pathological status of ALNs, number of metastatic ALNs, LVI, and perineural invasion status, were obtained from the patients' medical records. A pathological positive status of ALN metastasis was defined as macrometastasis (foci > 2.0 mm) or micrometastasis (foci 0.2–2.0 mm) identified by hematoxylin–eosin staining [7]. LVI was defined as the presence of tumor cells within the space of the endothelial cells [22]. The cutoff value for the positivity of HER-2, PR, and ER was used from internationally recognized standards [23, 24].

### MRI technique

All images were acquired by a 3 T MR system (Magnetom Skyra, Siemens Healthcare) using a 16-channel bilateral breast coil. Table E1 in the supplementary material summarizes the MRI acquisition parameters. The contrast agent gadopentetate dimeglumine (Bayer Pharmaceuticals Corporation) at a dose of 0.2 ml/kg was given by intravenous injection at a rate of 3.0 ml/s.

### Image analysis

MR image analyses were performed on the DCE software package (Tissue 4D, version: syngo MR D13, Siemens Healthcare), and DCE-derived parametric maps were automatically generated after motion correction. Breast tumors were identified on the DCE-MRI images, as the prominent enhancement area corresponded to the high signal area in the DWI image (b = 800 s/mm^2^) with a low signal area on the ADC map. Two radiologists (W.F. and F.C., with more than 15 years of experience) who were both blinded to the clinicopathological data reviewed the DCE-MRI images and ADC maps independently to extract the quantitative parameters of the tumors, including the volume transfer constant (K^trans^, min^−1^), reverse reflux rate constant (K_ep_, min^−1^), extracellular extravascular volume fraction (V_e_), rate of contrast enhancement for inflow (W-in, min^−1^), rate of contrast decay for outflow (W-out, min^−1^), time to peak enhancement after injection (TTP, min), and ADC value (× 10 ^−3^ mm^2^/s). The region of interest (ROI) drawing principles were as follows (Figure E1 in the supplementary material): ROIs with a minimum area of 0.10 cm^2^ were manually drawn on the continuous three maximum sections with the greatest enhancement areas of the tumors, avoiding visible blood vessels, obvious bleeding, and necrotic and cystic areas, with the same position and size on both the DCE-derived parametric maps and the ADC maps. The mean values of the parameters were taken as the final values. In addition, tumor sizes, including the maximum, minimum, and effective diameter of the tumor on the largest section, were recorded on the DCE-MRI images.

### Clinical treatment

All patients underwent surgical treatment after breast MRI. The surgical methods for BC include breast-conserving surgery, mastectomy, and breast reconstruction surgery, which were determined by the National Guidelines for the Diagnosis and Treatment of Breast Cancer 2022 in China (English version)[23]. Of 219 patients, 21 patients had received breast-conserving surgery, 189 patients had received mastectomy, and 9 patients had received breast reconstruction surgery. Furthermore, 171 patients received adjuvant chemotherapy after surgery, 71 received radiation therapy after surgery, and 121 received adjuvant endocrine therapy after surgery. Moreover, 43 patients had received all three treatment methods after surgery.

### Follow-up

All patients underwent clinical and imaging follow-up with chest CT, breast mammography, or breast MRI until progression, followed by routine follow-up until death. According to the Chinese Anti-Cancer Association guidelines for the diagnosis and treatment of breast cancer (2021 version), all patients were postoperatively followed up with a physical examination, laboratory tests, CT, mammography, and ultrasound every 3 months for the first two years, once every 6 months during years 3 to 5, and once a year after 5 years. Additionally, the patients with breast-conserving surgery were followed up with breast MRI once a year. Recurrence-free survival (RFS) was measured in months from the date of surgery to the first date of local recurrence, distant metastasis, or the last follow-up date, whichever came first. Overall survival (OS) was measured in months from the date of surgery to the date of death or the last follow-up date. The last follow-up date was March 1, 2021.

### Point-based scoring system development

A PSS was constructed using the method described by Sullivan et al. [25]. Continuous variables were transformed into binary variables according to the cutoff values determined by the largest Youden index to reduce the PSS score range and simplify its eventual clinical application. In the primary cohort, the cutoff values for age, maximum tumor diameter, minimum tumor diameter, effective tumor diameter, K^trans^, K_ep_, V_e_, W-in, W-out, TTP, and ADC were 48.5 years,3.55 cm,1.95 cm,3.13 cm,0.298 min^−1^,0.982 min^−1^,0.253, 0.674 min^−1^,-0.013 min^−1^,0.778 min, and 0.736 × 10^−3^ mm^2^/s, respectively. All variables were included in the multivariate logistic regression analysis following the backward stepwise selection method. The point of each selected variable was assigned based on its β-coefficient in the multivariate logistic regression analysis and rounded to the nearest 0.5. The final PSS score was obtained by summing the points of each variable. The odds ratios (ORs) with 95% confidence intervals (CIs) of each variable were also calculated.

### Statistical analysis

R version 3.6.0 was used for statistical analysis. Continuous variables with a normal distribution are expressed as the mean ± standard deviation, and categorical variables are expressed as counts and percentages. Data between the ALN metastasis and non-ALN metastasis groups were compared with Student's t test, chi-squared test, or Kruskal–Wallis H test, if appropriate. In addition, the intraclass correlation coefficient (ICC) was used to assess the interobserver agreement of the DCE-MRI parameters and the ADC values between the two radiologists [26, 27]. An ICC value between 0.61 and 0.80 indicates good agreement, while an ICC value ≥ 0.81 indicates excellent agreement [28]. A receiver operating characteristic (ROC) curve was created to estimate the discriminating power of the models, and the area under the curve (AUC), accuracy, sensitivity and specificity were calculated [27]. The ROCs of the models were compared by using the DelLong test. For the survival analysis, the patients were divided into low-risk and high-risk groups according to the PSS point, and the grouping threshold was determined by the X-tile method [29]*.* Kaplan–Meier curves for the high-risk and low-risk groups were plotted, and the prognosis of the high- and low-risk groups was compared by using the log rank test.* P* < 0.05 was considered statistically significant.

## Results

### Patient characteristics

Of 219 patients, 110 patients had non-ALN metastasis and 109 patients had ALN metastasis. The characteristics of the patients in the total, primary, and validation cohorts are listed in Table 1. The ALN metastasis positivity was 49.7% (76/153) and 50.0% (33/66) in the primary cohort and validation cohort, respectively. The patients with ALN metastasis had higher maximum, minimum, and effective tumor diameters, and higher T stages than the patients without ALN metastasis in the total, primary, and validation cohorts (all *P*** < **0.05). Compared to the patients without ALN metastasis, higher positive rates of LVI and lower V_e_ and W-out values were more pronounced in the patients with ALN metastasis in the total and primary cohorts (all *P* < 0.05), but these differences were not confirmed in the validation cohort (all *P* > 0.05). In addition, the patients with ALN metastasis had a higher Ki-67 index than patients without ALN metastasis in the total cohort (*P* = 0.038), but this was not confirmed in the primary and validation cohorts (all *P* > 0.05).

**Table 1:** Characteristics of patients with and without axillary lymph node metastasis

	Total cohort (*n* = 219)			Primary cohort (*n* = 153)			Validation cohort (*n* = 66)		
Characteristics	Non-ALN metastasis (*n* = 110)	ALN metastasis (*n* = 109)	*P*	Non-ALN metastasis (*n* = 77)	ALN metastasis (*n* = 76)	*P*	Non-ALN metastasis (*n* = 33)	ALN metastasis (*n* = 33)	*P*
Age (years)	51.85 ± 10.68	49.91 ± 11.13	0.191^a^	52.04 ± 10.71	51.13 ± 11.87	0.620^a^	51.39 ± 10.78	47.10 ± 8.74	0.080^a^
Maximum diameter (cm)	2.74 ± 1.03	3.63 ± 1.59	** < 0.001**^**a**^	2.73 ± 1.04	3.53 ± 1.54	** < 0.001**^**a**^	2.78 ± 1.01	3.87 ± 1.70	**0.003**^**a**^
Minimum diameter (cm)	1.80 ± 0.63	2.30 ± 1.02	** < 0.001**^**a**^	1.80 ± 0.63	2.28 ± 1.05	**0.001**^**a**^	1.81 ± 0.65	2.33 ± 0.99	**0.014**^**a**^
Effective diameter (cm)	2.27 ± 0.78	2.97 ± 1.22	** < 0.001**^**a**^	2.26 ± 0.79	2.91 ± 1.20	** < 0.001**^**a**^	2.30 ± 0.77	3.10 ± 1.27	**0.003**^**a**^
Perineural invasion			0.542^b^			0.479^b^			1.000^b^
Negative	105(95.45%)	102(93.58%)		74(96.10%)	70(92.11%)		31(93.94%)	32(96.97%)	
Positive	5(4.55%)	7(6.42%)		3(3.90%)	6(7.89%)		2(6.06%)	1(3.03%)	
LVI			**0.014**^**b**^			**0.034**^**b**^			0.202^b^
Negative	93(84.55%)	77(70.64%)		64(83.12%)	52(68.42%)		29(87.88%)	25(75.76%)	
Positive	17(15.45%)	32(29.36%)		13(16.88%)	24(31.58%)		4(12.12%)	8(24.24%)	
ER			0.959^b^			0.542^b^			0.306^b^
Negative	40(36.36%)	40(36.70%)		30(38.96%)	26(34.21%)		10(30.30%)	14(42.42%)	
Positive	70(63.64%)	69(63.30%)		47(61.04%)	50(65.79%)		23(69.70%)	19(57.58%)	
PR			0.453^b^			0.939^b^			0.138^b^
Negative	56(50.91%)	61(55.96%)		41(53.25%)	40(52.63%)		15(45.45%)	21(63.64%)	
Positive	54(49.09%)	48(44.04%)		36(46.75%)	36(47.37%)		18(54.55%)	12(36.36%)	
HER-2			0.848^b^			0.764^b^			0.438^b^
Negative	76(69.09%)	74(67.89%)		53(68.83%)	54(71.05%)		23(69.70%)	20(60.61%)	
Positive	34(30.91%)	35(32.11%)		24(31.17%)	22(28.95%)		10(30.30%)	13(39.39%)	
Ki-67			**0.038**^**b**^			0.117^b^			0.159^b^
< 20%	37(33.64%)	23(21.10%)		26(33.77%)	17(22.37%)		11(33.33%)	6(18.18%)	
≥ 20%	73(66.36%)	86(78.90%)		51(66.23%)	59(77.63%)		22(66.67%)	27(81.82%)	
T stage			** < 0.001**^**c**^			** < 0.001**^**c**^			**0.028**^**c**^
T1	49(44.55%)	21(19.27%)		35(45.45%)	14(18.42%)		14(42.42%)	7(21.21%)	
T2	58(52.73%)	70(64.22%)		41(53.25%)	51(67.11%)		17(51.52%)	19(57.58%)	
T3	1(0.91%)	11(10.09%)		0(0.00%)	5(6.58%)		1(3.03%)	6(18.18%)	
T4	2(1.82%)	7(6.42%)		1(1.30%)	6(7.89%)		1(3.03%)	1(3.03%)	
M stage			0.671^b^			0.239^b^			1.000^b^
M0	108(98.18%)	105(96.33%)		77(100.00%)	73(96.05%)		31(93.94%)	32(96.97%)	
M1	2(1.82%)	4(3.67%)		0(0.00%)	3(3.95%)		2(6.06%)	1(3.03%)	
AJCC stage			0.241^c^			0.615^c^			0.165^c^
I	22(20.00%)	12(11.01%)		15(19.48%)	10(13.16%)		7(21.21%)	2(6.06%)	
II	46(41.82%)	47(43.12%)		32(41.56%)	33(43.42%)		14(42.42%)	14(42.42%)	
III	18(16.36%)	30(27.52%)		12(15.58%)	19(25.00%)		6(18.18%)	11(33.33%)	
IV	24(21.82%)	20(18.35%)		18(23.38%)	14(18.42%)		6(18.18%)	6(18.18%)	
MRI parameters									
K^trans^ (min^−1^)	0.22 ± 0.12	0.19 ± 0.12	0.058^a^	0.22 ± 0.11	0.19 ± 0.11	**0.047**^**a**^	0.21 ± 0.14	0.20 ± 0.14	0.598^a^
K_ep_ (min^−1^)	0.82 ± 0.25	0.86 ± 0.22	0.269^a^	0.84 ± 0.24	0.85 ± 0.23	0.756^a^	0.79 ± 0.29	0.88 ± 0.20	0.144^a^
V_e_	0.27 ± 0.13	0.22 ± 0.12	**0.006**^**a**^	0.27 ± 0.12	0.22 ± 0.11	**0.022**^**a**^	0.29 ± 0.15	0.23 ± 0.15	0.115^a^
W-in (min^−1^)	0.57 ± 0.21	0.60 ± 0.27	0.368^a^	0.56 ± 0.19	0.59 ± 0.27	0.365^a^	0.59 ± 0.26	0.61 ± 0.28	0.775^a^
W-out (min^−1^)	-0.01 ± 0.02	-0.02 ± 0.02	**0.002**^**a**^	-0.01 ± 0.02	-0.02 ± 0.02	**0.009**^**a**^	-0.01 ± 0.02	-0.02 ± 0.02	0.081^a^
TTP (min)	0.67 ± 0.19	0.67 ± 0.20	0.983^a^	0.67 ± 0.19	0.68 ± 0.20	0.803^a^	0.67 ± 0.21	0.65 ± 0.21	0.698^a^
ADC (× 10 ^−3^ mm^2^/s)	0.87 ± 0.15	0.84 ± 0.13	0.158^a^	0.87 ± 0.14	0.84 ± 0.15	0.239^a^	0.86 ± 0.17	0.83 ± 0.09	0.447^a^

*Notes*. *P*^a^: Student’s t test, *P*^b^: chi-squared test,* P*^c^: Kruskal–Wallis H test

*Abbreviations*: *ALN* Axillary lymph node, *LVI* Lymphovascular invasion, *ER* Estrogen receptor, *PR* Progesterone receptor, *HER-2* Human epidermal growth factor receptor-2, *ADC* Apparent diffusion coefficient

### Agreement between two readers

The ICC values between the two readers for *K*^trans^, *K*_ep_, *V*_e_, W-in, W-out, TTP, and ADC were 0.995 (95% CI: 0.994–0.996), 0.775 (95% CI: 0.716–0.823), 0.988 (95% CI: 0.984–0.990), 0.990 (95% CI: 0.988–0.993), 0.793 (95% CI: 0.739–0.838), 0.965 (95% CI: 0.955–0.973), and 0.894 (95% CI: 0.863–0.917), respectively.

### Point-based scoring system development based on the primary cohort

Based on the backward stepwise selection method in the multivariate logistic regression analysis (Table 2), eight variables were associated with ALN metastasis and they were assigned scores for the final prediction rule: AJCC stage [II (1.0 points), III (17.5 points), IV (16.0 points)], T stage [T2 (1.0 points), T3 (17.0 points), T4 (2.0 points)], ER (positive,16.0 points), HER-2 (positive,1.5 points), maximum tumor diameter(≥ 3.55 cm,1.0 points), K_ep_ (≥ 0.982 min^−1^,1.0 points), V_e_ (≥ 0.253, -0.5 points), and TTP (≥ 0.778 min^−1^,1.0 points). The PSS point threshold for predicting the risk of ALN metastasis was 17.5, as obtained by the largest Youden index.

**Table 2:** Multivariate logistic regression analysis for predicting axillary lymph node metastasis with the assigned points

Variables	Multivariate regression			
	Odds Ratio (95% CI)	*P* value	β-Coefficient	Points
AJCC stage				
I	reference	-	-	0
II	2.28 (0.75 ~ 7.27)	0.151	0.825	1.0
III	37,836,559.51 (0 ~ NA)	0.988	17.448	17.5
IV	9,984,898.98 (0 ~ NA)	0.989	16.116	16.0
T stage				
T1	reference	-	-	0
T2	3.11 (1.3 ~ 8)	0.014	1.359	1.0
T3	27,337,664.44 (0 ~ NA)	0.990	17.123	17.0
T4	8.59 (0.83 ~ 205.36)	0.097	2.150	2.0
ER				
Negative	reference	-	-	0
Positive	10,968,914.8 (0 ~ NA)	0.989	16.21	16.0
HER-2				
Negative	reference	-	-	0
Positive	0.25 (0.07 ~ 0.84)	0.310	-1.388	-1.5
Maximum tumor diameter category (cm)				
< 3.55	reference	-	-	0
≥ 3.55	3.17 (1.26 ~ 8.45)	0.017	1.154	1.0
K_ep_ category (min^−1^)				
< 0.982	reference	-	-	0
≥ 0.982	2.50 (0.98 ~ 6.66)	0.059	0.918	1.0
V_e_ category				
< 0.253	reference	-	-	0
≥ 0.253	0.52 (0.24 ~ 1.13)	0.101	-0.652	-0.5
TTP category (min)				
< 0.778	reference	-	-	0
≥ 0.778	3.23 (1.27 ~ 8.74)	0.017	1.176	1.0

*Abbreviations*: *CI* Confidence intervals, *ER* Estrogen receptor, *PR* Progesterone receptor

### Performance of the point-based scoring system

**Fig. 2:**
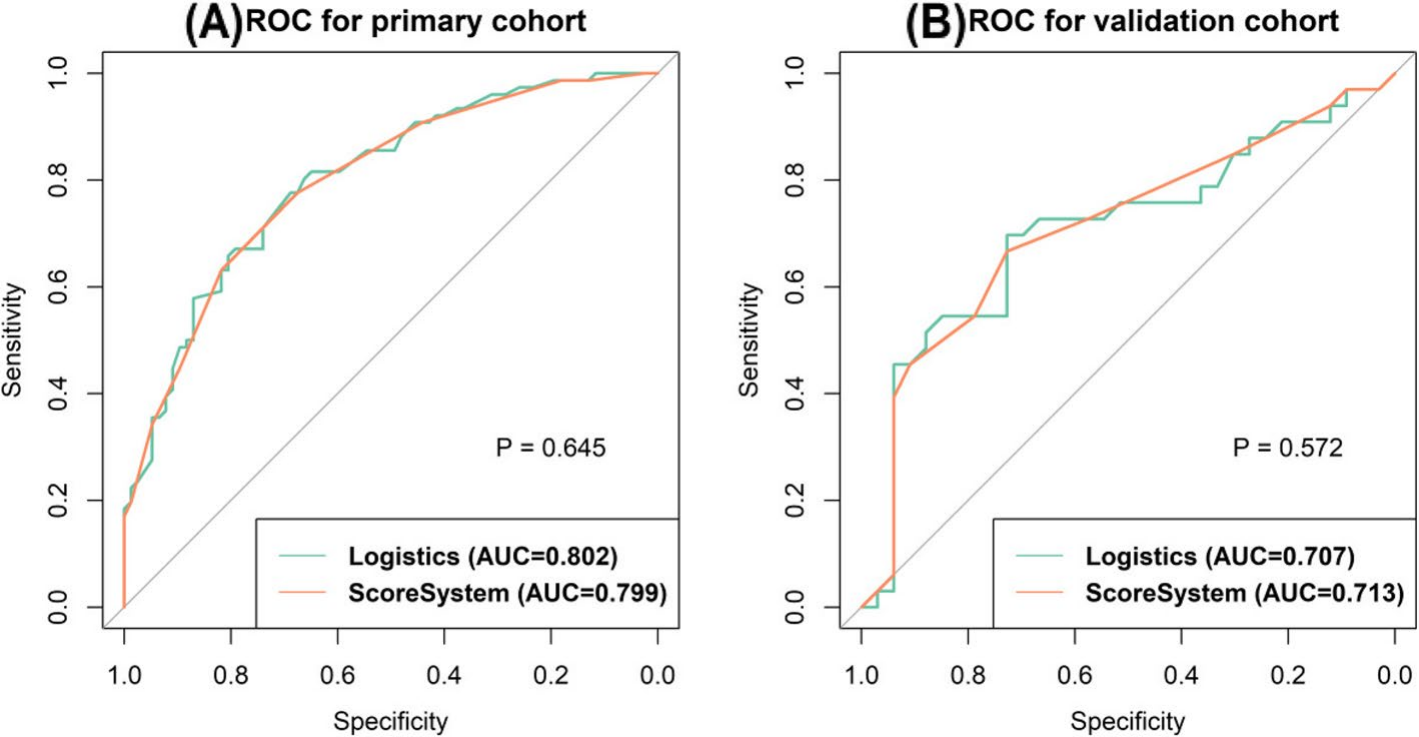
Receiver operator characteristic (ROC) curve for the prediction models in the primary cohort (**A**) and validation cohort (**B**)

**Fig. 3:**
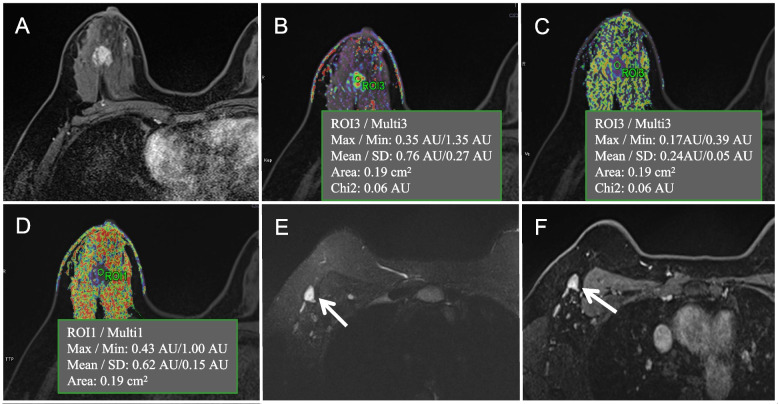
One example of the point-based scoring system for predicting breast cancer patients without axillary lymph node metastasis. **A** ~ **F** are MRI images of a female right breast cancer patient with invasive ductal carcinoma who was positive for human epidermal growth factor receptor-2, negative for estrogen receptor, AJCC stage III, and T2 stage. An axial contrast-enhanced T_1_WI image (**A**) shows a tumor with a maximum diameter of 2.3 cm located in the lower-outer quadrant. The mean values of K_ep_, V_e,_ and TTP from the corresponding pseudocolor images of K_ep_ (**B**), V_e,_ (**C**), and TTP (**D**) were 0.760 min^−1^, 0.190, and 0.620 min, respectively. Breast MRI was suspicious for axillary lymph node metastasis based on the following features: axial T_2_WI with fat suppression imaging (**E**) and axial contrast-enhanced T_1_WI imaging (**F**) showing cortical thickening, an oval shape and a long-to-short axis ratio of 1.4 for a maximal lymph node (white arrow) in axillary level I (nodes lateral and inferior to pectoralis minor muscle). However, the risk of axillary lymph node metastasis assessed by the scoring system was 17.0, with an axillary lymph node metastasis probability of 26.9%. The final postoperative pathology report showed that this tumor had no axillary lymph node metastasis

**Fig. 4:**
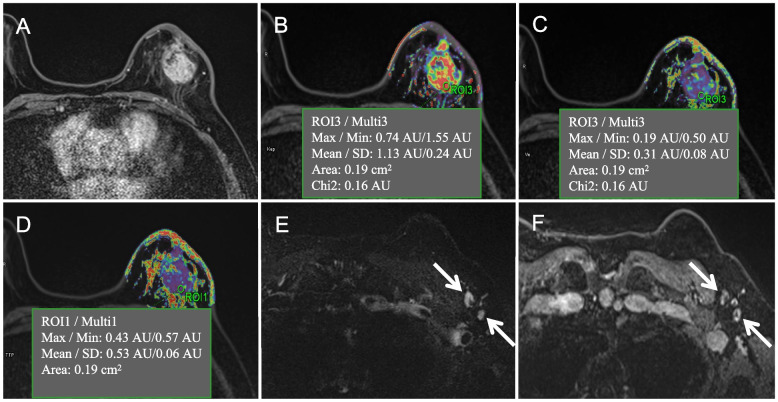
One example of the point-based scoring system for predicting breast cancer patients with axillary lymph node metastasis. **A** ~ **F** are MRI images of a female left breast cancer patient with invasive ductal carcinoma who was negative for human epidermal growth factor receptor-2, positive for estrogen receptor, AJCC stage II and T2 stage. An axial contrast-enhanced T_1_WI image (**A**) shows a tumor with a maximum diameter of 3.7 cm located in the upper-outer quadrant. The mean values of K_ep_, V_e,_ and TTP from the corresponding pseudocolor images of K_ep_ (**B**), V_e,_ (**C**), and TTP (**D**) were 1.130 min^−1^, 0.310, and 0.530 min, respectively. Breast MRI diagnosed no axillary lymph node metastasis for level I nodes due to the lack of all of the following features from axial T_2_WI with fat suppression imaging (**E**) and contrast-enhanced T_1_WI imaging (**F**): cortical thickening, missing fatty hilum, round shape, or a long-to-short axis ratio of less than 2 (long-to-short axis ratio was 2.2 for this patient). However, the risk of axillary lymph node metastasis assessed by the scoring system was 19.5,with an axillary lymph node metastasis probability of 81.8%.The final postoperative pathology report showed that this tumor had three axillary lymph node metastases at axillary level I (white arrow)

The logistic regression model that used all of the predictors for ALN metastasis obtained an AUC of 0.802 in the primary cohort and 0.707 in the validation cohort, and the PSS achieved an AUC of 0.799 in the primary cohort and 0.713 in the validation cohort (Table 3). ROC curve comparisons between the logistic regression model and the PSS are shown in Fig. 2. There were no significant AUC differences between the logistic regression model and PSS in either the primary cohort (*P* = 0.645) or in the validation cohort (*P* = 0.572). Table 4 presents the risk of ALN metastasis according to the PSS, and the ALN metastasis risk ranged from 4.7% to 100.0%. Figure 3 and Fig. 4 represent examples of the PSS in use.

**Table 3:** Performance of the prediction model for axillary lymph node metastasis

	Primary cohort				Validation cohort			
	AUC(95% CI)	Accuracy	Sensitivity	Specificity	AUC(95% CI)	Accuracy	Sensitivity	Specificity
Logistic regression model	0.802(0.733–0.871)	0.732	0.816	0.649	0.707(0.576–0.838)	0.712	0.697	0.727
PSS	0.799(0.731–0.868)	0.725	0.776	0.675	0.713(0.585–0.840)	0.697	0.667	0.727

*Abbreviations*: *PSS* Point-based scoring system, *CI* Confidence intervals

**Table 4:** Axillary lymph node metastasis risk according to the point-based scoring system

Risk score	ALN metastasis risk	Total number	Number of ALN metastasis
15	4.7%	4	1
15.5	7.6%	11	1
16	11.9%	6	1
16.5	18.2%	35	9
17	26.9%	41	14
17.5	37.8%	29	13
18	50.0%	26	18
18.5	62.2%	19	11
19	73.1%	17	13
19.5	81.8%	5	4
20	88.1%	3	3
20.5	92.4%	3	3
31	100%	1	1
33	100%	1	1
33.5	100%	2	2
34	100%	4	4
34.5	100%	1	1
35	100%	3	3
35.5	100%	3	2
36	100%	1	1
49.5	100%	1	1
50	100%	2	1
52.5	100%	1	1
Total	49.8%	219	109

*Note*. *ALN* Axillary lymph node

### Predictors of survival

All patients had completed RFS follow-up, and the median RFS of all patients was 32 [interquartile range (IQR):23.0–44.0] months, of which 26 patients had tumor recurrence and 193 patients had no tumor recurrence. The recurrence rate was 19.3% in the ALN metastasis group and 4.5% in the non-ALN metastasis group. In addition, 9 patients with tumor recurrence were deceased and 2 patients without tumor recurrence were deceased. While 218 patients had completed OS follow-up, the median OS of all patients was 43.8 (IQR:32.0–55.0) months, of which 11 patients were deceased. Of note, the overall death rate was 8.3% in the ALN metastasis group and 1.8% in the non-ALN metastasis group.

**Fig. 5:**
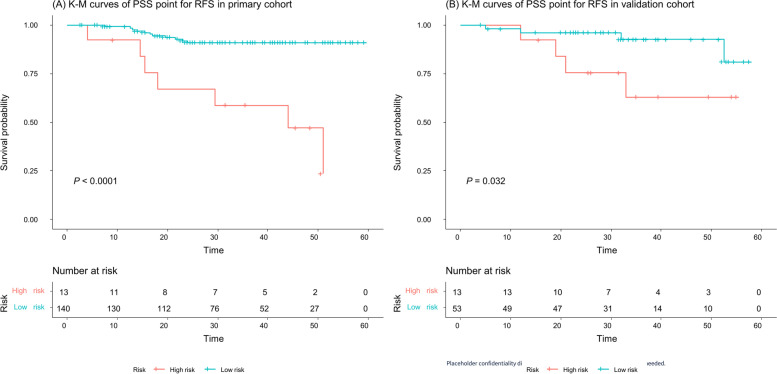
Kaplan–Meier recurrence-free survival (RFS) analyses of the low-risk and high-risk groups in the PSS. The high-risk group patients had a significantly worse RFS than the low-risk groups patients in both the primary cohort (**A**) and validation cohort (**B**)

**Fig. 6:**
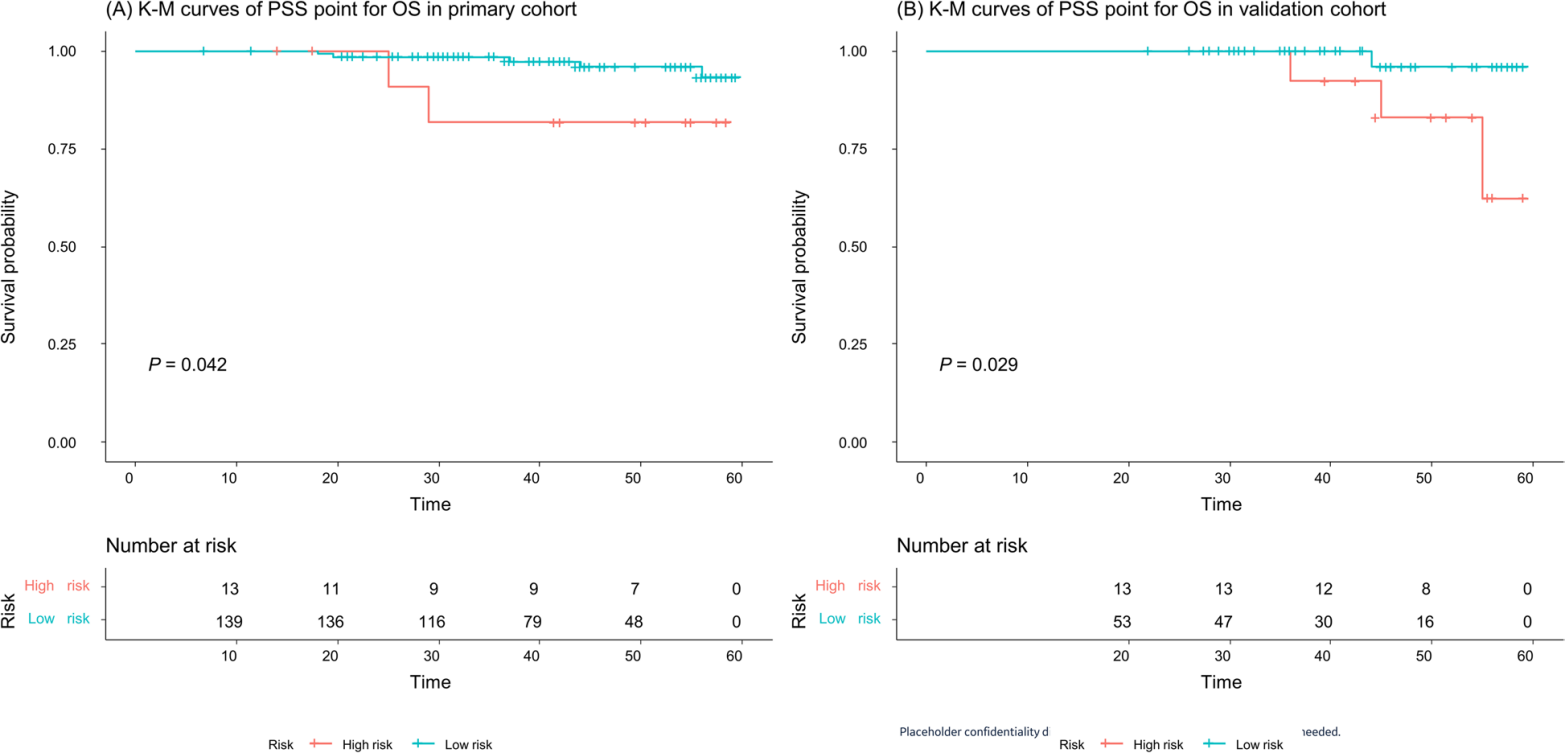
Kaplan–Meier overall survival (OS) analyses of the low-risk and high-risk groups in the PSS. The high-risk group patients had a significantly worse OS than the low-risk group patients in both the primary cohort (**A**) and validation cohort (**B**)

The PSS point threshold for the low-risk group and high-risk group was 19.5, as obtained by the X-tile method [29]. The median RFS was 32.0 (IQR:15.5–46.0) months for the high-risk group (> 19.5 points) and 32.5 (IQR:22.9–44.3) for the low-risk group (≤ 19.5 points) in the primary cohort (the comparative* P* value for the two survival curves using the log-rank test was lower than 0.0001, hereinafter, Fig. 5A) and 32.0 (IQR:21.0–40.0) months for the high-risk group and 32.0 (IQR:23.5–40.0) for the low-risk group in the validation cohort (*P* = 0.032, Fig. 5B). The Kaplan–Meier survival analysis showed that the median OS was 43.3 (IQR:31.9–56.0) months for the high-risk group and 50.0 (IQR:29.0–55.5) for the low-risk group in the primary cohort (*P* = 0.042, Fig. 6A) and 43.5 (IQR:32.0–55.0) months for the high-risk group and 50.5 (IQR:45.0–55.0) for the low-risk group in the validation cohort (*P* = 0.029, Fig. 6B).

## Discussion

In the present study, we developed and validated a PSS for stratifying the ALN metastasis risk of BC based on the combination of clinical features and quantitative MRI features and investigated the prognostic effect of the PSS. Our results showed that the PSS has good discrimination for stratifying the ALN metastasis risk of BC in the primary cohort and in the validation cohort. In addition, the RFS and OS of the high-risk group (> 19.5 points) patients were significantly lower than those of the low-risk (≤ 19.5 points) group patients in the PSS.

Risk stratification scoring systems have been used to guide clinical decision-making [16, 30–33]. A recent study conducted by MY et al. developed a scoring system to stratify the risk of lymphovascular invasion in BC patients with an AUC of 0.824 [16]. Another study from Ouldame et al. used a scoring system to predict axillary response after neoadjuvant chemotherapy in initially node-positive women with BC [34]. However, little is known about the ALN based on this scoring system. In this study, to develop the risk stratification scoring system, a multivariable logistic regression analysis was used to select the risk factors for ALN metastasis. The PSS point threshold for predicting the risk of ALN metastasis is 17.5 and is very easy for clinicians to use. In terms of assessing ALN metastasis, the scoring system showed good performance in the primary (AUC = 0.799) and validation cohorts (AUC = 0.713), which was similar to the result from Yang et al. [35]. Contrary to previously developed models that might be time-consuming and hard to apply, this risk stratification scoring system provides an easy tool for surgeons to assess breast cancer patients' risk for ALN metastasis prior to surgery [7, 10, 36, 37].

In this study, eight candidates, including AJCC stage, T stage, ER status, HER-2 status, maximum tumor diameter, K_ep_, V_e_, and TTP, were identified as risk factors for ALN metastasis from a stepwise logistic regression model. Our results showed that the selected variables in the scoring system were in line with those of the results reported in previous studies. For instance, Chen et al. found that HER-2 and tumor size were related to breast cancer ALN metastasis [38]. Yang et al. reported that ER and T stage were significantly associated with ALN status [35]. Ya et al. showed the ability of dynamic contrast-enhanced MRI parameters of K_ep_ and TTP to predict ALN metastasis in breast cancer patients [39]. Among the risk factors in this study, clinicopathologic risk factors for AJCC stage, T stage, ER, and HER-2 were more important than the MRI quantitative parameters of maximum tumor diameter, K_ep_, V_e_, and TTP based on their β-coefficient results in the logistic regression model. These results were consistent with previous results, in which clinicopathological risk factors were better predictors than imaging biomarkers [18, 40]. In addition, K_ep_ plays a more important role in the PSS than the parameters of V_e_ and TTP. The possible explanations may be as follows: K_ep_ may be less sensitive to the absolute value of the contrast agent concentration compared with V_e_ and TT. Another reason could be due to the complexity of the complex pathological characteristics of BC, which were consistent with previous results [28, 41].

The determination of ALN metastasis has important clinical significance for patients' overall recurrence and survival [3, 42]. Generally, patients with ALN metastasis have poor outcomes and a higher risk of local recurrence and distant metastasis than patients without ALN metastasis. Similar results were observed in our study: the patients with ALN metastasis had a higher recurrence rate and overall death rate than the patients without ALN metastasis (recurrence rate:19.3% vs. 4.5%, and death rate 5.0% vs. 1.8%, respectively). To explore prognostic significance of the PSS, we compared the prognosis of high- and low-risk groups in the PSS. The Kaplan–Meier survival analysis showed that the median RFS and OS of the high-risk group patients were significantly lower than those of the low-risk group patients in both the primary cohort and validation cohort, suggesting that the PSS can be routinely used in predicting the prognosis of BC patients.

This study has several limitations. First, the PSS was developed based on the combination of clinicopathological and MRI features of BC instead of the ALN itself because it is difficult to match the biopsied or dissected ALN on MRI imaging. Second, these retrospective study results were obtained in a single institution with some inherent limitations and the results should be validated in a multicenter study. Third, some patients were followed up for less than 5 years, which partially affected the strength of prognostic information.

## Conclusion

In conclusion, a simple and reliable point-based scoring system can be used to stratify the ALN metastasis risk of BC. PSS greater than 19.5 was confirmed to be a predictor of short RFS and OS.

## Supplementary Information

**Additional file 1: Table E1.** Shows the MRI acquisition parametersAQ. **Figure E1.** Measurement of MRI quantitative parameters of tumors in a right breast cancer patient with invasive ductal carcinoma.

## Data Availability

The data cohorts used and/or analyzed in the present study are available from the corresponding authors upon reasonable request.

## Abbreviations

ALN: Axillary lymph node
BC: Breast cancer
ER: Estrogen receptor
PR: Progesterone receptor
HER-2: Human epidermal growth factor receptor-2
PSS: Point-based scoring system
RFS: Recurrence-free survival
OS: Overall survival
