# Supplementary material for: Development and validation of a point-based scoring system for predicting axillary lymph node metastasis and disease outcome in breast cancer using clinicopathological and multiparametric MRI features
Source: Cancer Imaging. 2023 Jun 1;23:54. doi: 10.1186/s40644-023-00564-9 (PMC10233973; doi:10.1186/s40644-023-00564-9)
Supplement: Supplementary file 1 — Additional file 1: Table E1. Shows the MRI acquisition parametersAQ. Figure E1. Measurement of MRI quantitative parameters of tumors in a right breast cancer patient with invasive ductal carcinoma. [file 40644_2023_564_MOESM1_ESM.docx]

**Supplementary Materials**

**Table of Contents:**

[1 MRI acquisition parameters (Table E1) . 2](#a)

[2 One example of the measurement of the MRI quantitative parameters (Figure E1) 3](#b)

# MRI acquisition parameters (Table E1)

Conventional MR examinations including a T1-weighted image and a T2-weighted image were acquired. DWI was performed using a readout segmented echo planar imaging technique with fat suppression in the transverse plane prior to DCE-MRI. DCE-MRI was performed using a combination of volume interpolated breath hold examination with view sharing time resolved imaging with interleaved stochastic trajectories sequence. **Table E1** shows the MRI acquisition parameters.

| **Table E1** Technical parameters | | | | |
| --- | --- | --- | --- | --- |
| Parameters | DWI | DCE-MRI | T_1_WI | T_2_WI |
| TR (ms) | 4800 | 6.4 | 6.4 | 3570 |
| TE (ms) | 56/86 | 3.34 | 3.3 | 74 |
| TI (ms) | - | - | - | 230 |
| FOV (mm) | 170×340 | 340×340 | 288×384 | 341×341 |
| Matrix | 96×192 | 288×384 | 288×384 | 314×448 |
| Slice thickness (mm) | 4.0 | 2.0 | 2.0 | 4.0 |
| total slices | 50 | 80 | 112 | 34 |
| Slice gap (mm) | 0.8 | no slice gap | 0.4 | 0.8 |
| Flip angle | 180° | 9° | 2° | 8° |
| Total acquisition time (s) | 298 | 305 | 60 | 84 |
| Temporal resolution (s) | - | 8.7 | - | - |
| b-value (s/mm^2^) | 50 and 800 | - | - | - |

**2. One example of the measurement for the MRI quantitative parameters (Figure E1)**

**Figure E1** presents one example of the evaluation of MRI qualitative features from a breast cancer patient with invasive ductal carcinoma.


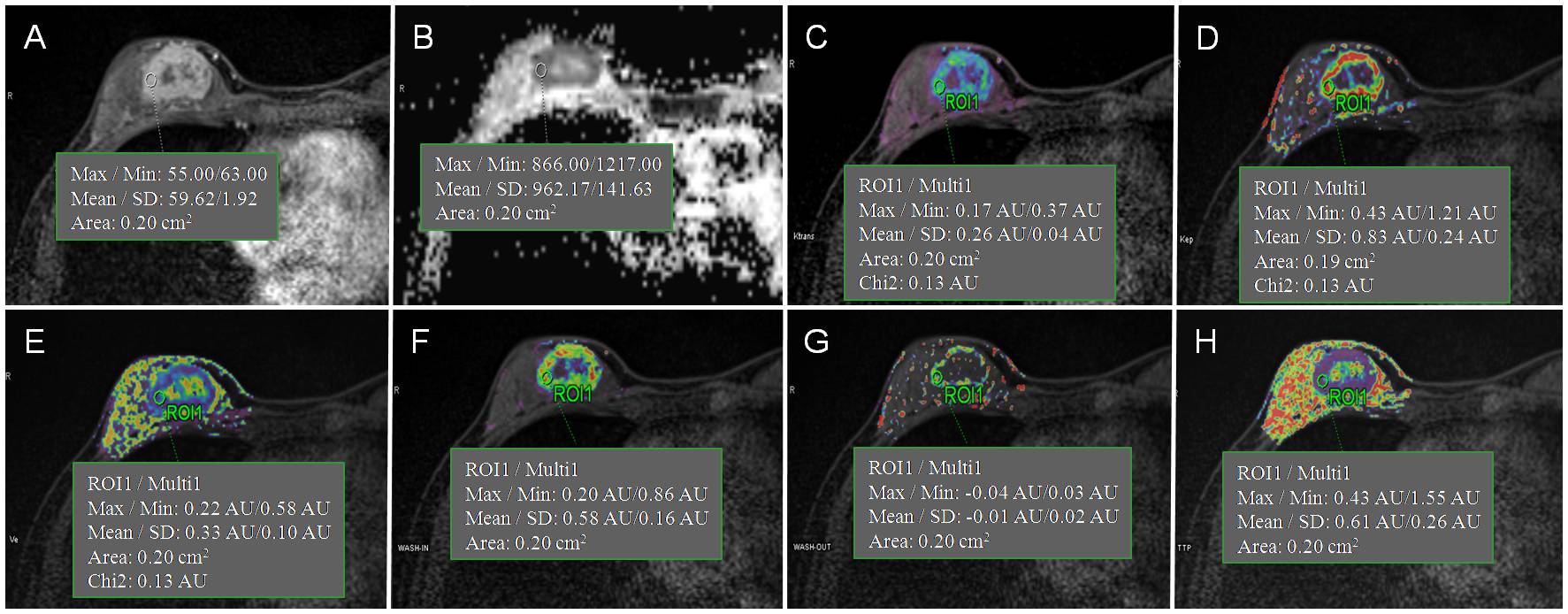


**Figure E1** Measurement of MRI quantitative parameters of tumors in a right breast cancer patient with invasive ductal carcinoma. With the axial contrast-enhanced T_1_WI image (A) as a reference, the regions of interests (ROIs) with a minimum area of 0.10 cm^2^ were manually drawn on the maximal section with the greatest enhancement areas of tumors on the ADC maps and DCE-derived parametric maps, with the same size and position. Visible blood vessels, obvious bleeding, and necrotic and cystic areas should not be involved in the ROIs. The mean values of ADC (B), K^trans^ (C), K_ep_ (D), V_e_ (E), W-in (F), W-out (G), and TTP (H) from the corresponding maps were 0.962 ×10^-3^ mm^2^/s, 0.260 min, 0.830 min, 0.330, 0.580 min^-1^, -0.01 min^-1^, and 0.610 min, respectively.
